# Improvement of Mixed Inflammatory Environment in Nasal Secretions of Diffuse Type 2 Chronic Rhinosinusitis With Nasal Polyps Under Dupilumab

**DOI:** 10.1002/clt2.70180

**Published:** 2026-06-03

**Authors:** Fabio S. Ryser, Tina Mauthe, Catrin Brühlmann, Michael B. Soyka, Urs C. Steiner

**Affiliations:** ^1^ Graduate School for Health Sciences University of Bern Bern Switzerland; ^2^ Department of Rheumatology and Immunology Inselspital, Bern University Hospital University of Bern Bern Switzerland; ^3^ Department of Oto‐Rhino‐Laryngology and Head Neck Surgery University and University Hospital Zurich Zürich Switzerland; ^4^ Department of Clinical Chemistry Inselspital Bern University Hospital University of Bern Bern Switzerland

**Keywords:** biologicals, biomarker, CRSwNP, endotype, nasal secretion, type 2 inflammation

## Abstract

**Background:**

In Western countries, Chronic Rhinosinusitis with Nasal Polyps (CRSwNP) is predominantly associated with a type 2 inflammatory endotype. While nasal secretion analysis shows promise for disease endotyping, current biomarkers remain limited for accurate stratification, disease monitoring, and prediction of treatment response.

**Objective:**

This study aimed to investigate the inflammatory patterns in nasal secretions of patients with type 2 Chronic Rhinosinusitis with Nasal Polyps (CRSwNP) undergoing treatment with dupilumab, an anti‐IL4Rα antibody. A second objective was to evaluate markers for therapy response and monitoring.

**Methods:**

Nasal secretions and blood samples were collected at four time points from 23 patients with histological type 2 CRSwNP undergoing dupilumab treatment and compared with 10 healthy controls. Samples were analysed using proximity extension assay method by Olink.

**Results:**

Proteomic analysis of nasal secretions revealed 50 differentially expressed proteins in type 2 CRSwNP compared to healthy controls. Nasal secretions of most patients revealed a mixed inflammatory endotype. Under dupilumab treatment, cytokines of all different endotypes decreased. The cytokines with the highest mean change (MCP‐4, CCL23, GDNF and CCL11) showed good associations with clinical variables and were effective predictors of dupilumab response and disease control.

**Conclusion:**

The inflammatory environment of the nasal mucosa in histological type 2 CRSwNP is most often characterized by a mixed type 1, 2, and 3 inflammatory endotype. Targeting type 2 inflammation with dupilumab reduces inflammation markers of all three types of inflammation. GDNF has emerged as a valuable marker for stratifying therapy and monitoring success in type 2 CRSwNP under dupilumab treatment.

**Abbreviations**
AUC: area under the curve
CCL: CC‐chemokine ligand
CRS: chronic rhinosinusitis
CRSwNP: chronic rhinosinusitis with nasal polyps
CXCL: CXC‐chemokine
DEP: differentially expressed proteins
EPOS20: European position paper on rhinosinusitis and nasal polyps 2020
EUFOREA: European forum for research and education in allergy and airway diseases
FeNO: fractional exhaled nitric oxide
GDNF: glial cell line‐derived neurotrophic factor
IFN‐γ: interferon‐γ
IL: interleukin
ILC: innate lymphoid cell
IQR: interquartile range
NERD: NSAID‐exacerbated respiratory disease
NET: neutrophil extracellular traps
NPS: nasal polyp score
NPX: normalized protein expression
NSAID: nonsteroidal anti‐inflammatory drugs
PEA: proximity extension assay
ROC: receiver operating curve
SNOT‐22: Sino‐Nasal Outcome Test 22
SSIT‐12: Sniffin’Sticks‐12 identification test
Th: T‐helper cell

## 1 Introduction

Chronic Rhinosinusitis (CRS) is a common chronic inflammatory disease of the sinonasal mucosa [1, 2]. According to endoscopic findings, CRS is categorized phenotypically as either CRS with nasal polyps (CRSwNP) or CRS without nasal polyps (CRSsNP) [3]. Symptoms of CRSwNP include loss of smell, nasal congestion, headaches, rhinorrhea and fatigue. It is often difficult to treat and can have a significant impact on quality of life [4, 5].

In Western countries, CRSwNP is most commonly associated with a type 2 inflammatory endotype, mediated by T‐helper (Th) 2cells, innate lymphoid cells type 2 (ILC2), eosinophilic granulocytes, and the corresponding cytokines IL‐4, IL‐5 and IL‐13. However, also type 1 inflammatory cells and mediators characterized by Th1 T‐cells, ILC1, cytotoxic lymphocytes, and the cytokines IFN‐γ, CXCL9 and CXCL11 as well as type 3 inflammation, characterized by Th17 T‐cells, ILC3 cells, neutrophilic granulocytes, and cytokines including IL‐17A, IL‐1β, IL‐8, CXCL1, CXCL2, CCL20 can be associated with so called type 2 predominant CRSwNP. Approximately one‐quarter of patients with type 2 CRSwNP exhibit a mixed type 1, type 2 and type 3 inflammatory endotype [6, 7, 8, 9, 10, 11].

According to the EPOS20 criteria, type 2 CRSwNP is determined by biopsy of the nasal mucosa with > 10 eosinophils/high power field or serologic markers like blood eosinophilia or high IgE. Treatment response and disease control are assessed using the nasal polyp score (NPS), Sino‐Nasal Outcome Test 22 (SNOT‐22), and Sniffin’Sticks‐12 identification test (SSIT‐12) as well as disease specific control charts (3, 9). As diagnosis and monitoring of type 2 CRSwNP are demanding, non‐invasive methods such as nasal secretion analysis for diagnosis, therapy stratification and disease monitoring would be desirable. It has been demonstrated that protein expressions of nasal secretion in patients with type 2 CRSwNP largely corresponds to those in nasal mucosal tissue [7, 8, 12, 13, 14].

Dupilumab, an anti‐IL4Rα‐monoclonal antibody, is a highly effective therapy for type 2 CRSwNP [15, 16]. In addition to reducing type 2 inflammation, it contributes to the restoration of the epithelial barrier and the microbial dysbiosis in patients with a type 2 inflammation such as CRSwNP or atopic dermatitis [17, 18, 19, 20, 21].

The aim of this study was to investigate different inflammatory patterns of nasal secretion in type 2 CRSwNP (referred to as secretory endotypes) and changes of the inflammatory endotypes in nasal secretions and blood under therapy with dupilumab. A second aim was to evaluate markers in nasal secretions that could provide information about therapy response and disease control in type 2 CRSwNP patients.

## 2 Methods

### 2.1 Patients and Study Design

This is a multi‐centric prospective observational case‐control study conducted at the Department of Otorhinolaryngology, Head and Neck Surgery, at the University Hospital Zurich, Zurich, Switzerland, the Department of Immunology, University Hospital Zurich, Zurich, Switzerland and the Department of Rheumatology and Immunology, Inselspital, University Hospital Bern, Bern, Switzerland. The study was approved by the cantonal Ethics Committees (KEK‐2021‐01213) of Bern and Zurich, Switzerland. The study was conducted according to the Declaration of Helsinki. Patients were screened and enrolled between December 2021 and November 2022 and signed the informed consent prior to inclusion and sampling.

The study included 23 patients suffering from uncontrolled diffuse type 2 CRSwNP according to EPOS20 criteria. Type 2 inflammatory endotype was determined by biopsy of the nasal mucosa (> 10 eosinophils/high power field) [3]. All participants started dupilumab (Dupixent by Sanofi and Regeneron Pharmaceuticals Inc.) therapy 300 mg subcutaneously every other week as per Swiss indication criteria (bilateral nasal polys, at least 1 sinonasal surgery, SNOT‐22 ≥ 50 points, nasal polyp score ≥ 5, sustained symptoms for at least 12 weeks including nasal congestion, hyposmia, and rhinorrhea). The treatment was not part of this study. The first injection was conducted at the Department of Immunology, University Hospital Zurich including an introduction for follow‐up self‐administering at home. Appropriate medical therapy with nasal steroids (0.05 mg mometason furoat) twice two sprays in each nostril and saline rinses daily was continued throughout the study according to EPOS20 recommendations. No participants received oral corticosteroids 3 months prior to inclusion. Parts of the study results of this cohort were published previously with other research questions and targets [17, 22].

### 2.2 Controls

As controls, 10 healthy individuals without asthma, atopic dermatitis and without any infections and symptoms of allergy at time of sampling were recruited. All participants signed the informed consent.

### 2.3 Specimen Sampling

Nasal secretion and blood samples were collected on day 0 (23 samples), day 28 (19 samples), day 90 (18 samples), and day 180 (17 samples) after start of dupilumab therapy. At time of sampling, patients with ongoing infections were excluded as well as nasal secretions samples containing blood. For nasal secretion, one surgical patty (½″ × 1″) by Codman (Integra LifeSciences Corporation, Mansfield USA) was placed between the septum and the inferior turbinate of each nostril for 10 min using a nasal speculum and tweezers. The patties were then removed. Assay diluent, 200 μL 0.9% NaCl was added to each patty. The patties were centrifuged for 5 min for 1000 U/min in a corning tube. After centrifuging, the secretions were pipetted into two Eppendorf Conical Tubes and stored at −80°C until further analysis.

At each visit, blood samples were collected in 7.5 mL serum tubes, then centrifuged immediately and stored at −80°C in the laboratory of the Department of Oto‐Rhino‐Laryngology and Head and Neck Surgery, University Hospital Zurich.

### 2.4 Proximity Extension Assay

Eighty‐eight blood samples and 88 nasal secretion samples were sent to the Swiss Institute of Allergy and Asthma Research in Davos, Switzerland for Olink analysis. The samples were randomized within the plate prior to analysis according to manufacturer’s instructions. Protein concentrations of 92 proteins were measured using the Proximity Extension Assay (PEA) technology with the Olink Target 96 Inflammation panel (Table S1). The measurements were conducted according to the manufacturer's instructions. The values were normalized after measurement and reported in normalized protein expression units (NPX). NPX values from nasal secretions and blood were paired for subsequent statistical analysis. Mean changes were assessed using the NPX values. Some proteins were measured below the Limit of Detection (LOD) and therefore excluded for further analysis (β‐NGF, FGF‐5, IL‐2, IL‐20, and IL‐2RB).

### 2.5 Nasal Secretion Endotyping

Inflammatory endotypes 1, 2, and 3 were defined by IFN‐γ, IL‐5, and IL‐17A concentrations respectively, exceeding the 95th percentile of control nasal‐secretion levels as recently published [6, 23]. Patients with elevations in more than one signature cytokine were classified as a mixed endotype, whereas those without any elevated signature cytokines were classified as undefined. Because dupilumab directly targets the IL‐4‐ and IL‐13 receptors these two cytokines could not be used as markers.

### 2.6 Statistical Analysis

Patient characteristics and demographics were reported in mean or median and standard deviation or interquartile range (IQR) according to parametric or non‐parametric data. Longitudinal clinical data was tested using paired Wilcoxon signed‐rank test, Mood's mediant test or paired *t*‐test. PEA results were analysed using OlinkAnalyze package for R. We performed *t*‐test and paired *t*‐test. Correlation analyses were conducted using Spearman's correlation. Univariate linear regression was used to assess association between proteins and clinical variables. The biomarkers were further evaluated using univariate and multivariate logistic regression, receiver‐operating curve (ROC) and area under the curve (AUC). The analyses were post‐hoc corrected for multiple testing using Benjamini‐Hochberg corrections. All statistical analyses were calculated using R‐Studio and R (IBM Version 4.3.3). A *p* value of less than 0.05 was assumed to be significant.

## 3 Results

We analysed nasal secretion samples of 33 participants in total. Twenty‐three patients with CRSwNP and 10 healthy controls. Five nasal samples containing blood were excluded. Twenty‐one patients suffering from type 2 CRSwNP were also diagnosed with Asthma, nine patients had concomitant Non‐Steroidal‐Antiinflammatory‐Drug (NSAID)‐exacerbated respiratory disease (NERD) and 15 suffered additionally from allergies (Table 1).

**TABLE 1:** Patient and control demographics.

	CRSwNP under dupilumab (*N* = 23)	Healthy controls (*N* = 10)
Sex		
Male	18 (78.3%)	1 (10%)
Female	5 (21.7%)	9 (90%)
Age (mean, SD)	53.7 (11.5)	46.9 (10.7)*
Timepoint		
Day 0	23 (100%)	
Day 28	19	
Day 90	18	
Day 180	17	
Asthma	21 (91.3%)	0 (0%)
NERD	9 (39.1%)	0 (0%)
Allergy	15 (65.2%)	1 (10%)
Response to dupilumab		
Response	20 (87.0%)	
No response	2 (8.7%)	
Missing	1 (4.3%)	

Abbreviations: NERD, NSAID‐Exacerbated Respiratory Disease; SD, Standard Deviation.

^*^ *p* < 0.05.

Treatment response to biological treatment was defined according to EPOS2020/EUFOREA expert as an improvement in the SNOT‐22 of ≥ 12 points according to the MCID and a decrease of the NPS of ≥ 1 point or increase of the SSIT‐12 of ≥ 2 points and above 7 points at 3 months or 6 months after treatment start. Disease control was defined with a SNOT‐22 score below < 40 points, a total nasal polyp score of ≤ 2 points or hyposmia with a SSIT‐12 score of ≥ 7 points [24]. Twenty patients responded to dupilumab treatment. The non‐responders had persistently high SNOT‐22 scores (> 40) without meeting the MCID despite a NPS improvement of > 1 point.

As shown in Table 2, SNOT‐22, NPS, and SSIT‐12 scores improved rapidly during dupilumab treatment. By day 180, 94.1% of patients met criteria for disease control, defined as a SNOT‐22 score < 40 together with either a total NPS ≤ 2 or an SSIT‐12 score ≥ 7 [24, 25].

**TABLE 2:** Patient characteristics.

	Day 0 (*N* = 23)	Day 28 (*N* = 19)	Day 90 (*N* = 18)	Day 180 (*N* = 17)
SNOT‐22 score				
Mean (SD)	48.2 (17.8)	24.3 (17.8)**	17.7 (16.8)**	10.3 (14.8)**
Median [min, max]	48.0 [22.0, 79.0]	20.0 [0, 58.0]**	11.0 [0, 52.0]**	4.00 [0, 51.0]**
Missing	8 (34.8%)	4 (21.1%)	4 (22.2%)	2 (11.8%)
NPS				
Mean (SD)	5.48 (1.62)	4.06 (1.82)**	2.94 (2.04)**	1.82 (1.33)**
Median [min, max]	6.00 [1.0, 8.0]	4.00 [0, 6.00]**	3.00 [0, 6.0]**	2.00 [0, 4.0]**
Missing	0 (0%)	2 (10.5%)	0 (0%)	0 (0%)
Eosinophils				
Mean (SD)	0.421 (0.239)	0.534 (0.513)^ns^	0.275 (0.145)*	0.526 (0.554)^ns^
Median [min, max]	0.35 [0.11, 0.84]	0.32 [0.06, 1.74]^ns^	0.29 [0.05, 0.56]*	0.305 [0.04, 2.0]^ns^
Missing	2 (8.7%)	2 (10.5%)	1 (5.6%)	1 (5.9%)
12‐item Sniffin’ stick test				
Mean (SD)	5.09 (2.94)	6.44 (3.79)^ns^	9.00 (2.87)**	9.35 (2.62)**
Median [min, max]	5.00 [1.00, 11.0]	6.50 [1.00, 12.0]^ns^	10.0 [3.00, 12.0]**	10.0 [2.00, 12.0]**
Missing	1 (4.3%)	1 (5.3%)	0 (0%)	0 (0%)
Disease control				
Uncontrolled	20 (87.0%)	10 (52.6%)*	4 (22.2%)**	1 (5.9%)**
Controlled	3 (13.0%)	8 (42.1%)*	14 (77.8%)**	16 (94.1%)**
Missing	0 (0%)	1 (5.3%)	0 (0%)	0 (0%)

Abbreviations: FeNO, Fraction of expiratory nitrogen monoxide; NPS, Nasal Polyp Score; SNOT‐22, Sino‐Nasal‐Outcome Test Version 22.

FDR‐adjusted *p* values compared to day 0: ns = not significant, * < 0.05, ** < 0.01.

### 3.1 Protein Expression in Nasal Secretion of Patients With Type 2 CRSwNP Versus Healthy

**FIGURE 1:**
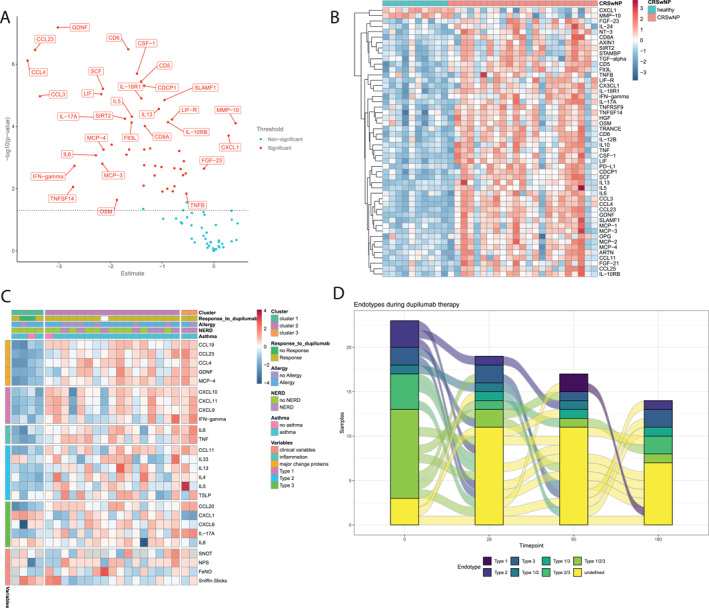
Nasal secretions protein expression in CRSwNP compared to healthy controls. (A) Volcano plot of differentially expressed proteins of CRSwNP day 0 (before dupilumab treatment start) and healthy controls with mean change (estimate) on *x*‐axis and –log10(*p* value) on *y*‐axis. Red dots indicate significant values. Reference group is CRSwNP. Thresholds are set at > 0.5 for log2 fold‐change and at < 0.05 for adjusted *p* values. (B) Heatmap of all 50 significant DEPs in CRSwNP day 0 compared to healthy controls. Values are plotted according to standardized *z*‐score. (C) Heatmap of proinflammatory, major change, Th1, Th2, Th17 cytokines at baseline in the different clusters with clinical parameters and comorbidities (response to dupilumab therapy, allergy, asthma, NERD). (D) Sankey plot of inflammatory endotypes (type 1, type 2, type 3, or mixed) at each time point during dupilumab therapy, assessed by cytokine levels in nasal secretions. Endotypes were defined as elevated IFN‐γ (type 1), IL‐5 (type 2), or IL‐17A (type 3) above the 95th percentile of controls.

The Olink analysis revealed 50 differential expressed proteins (DEP) in nasal secretion of patients with type 2 CRSwNP compared to healthy individuals. The two proteins CXCL1 and MMP‐10 were expressed at lower levels. Forty‐eight proteins were overexpressed compared to healthy. The proteins with the highest mean change were CCL23 (mean change 3.463, adj *p* value < 0.001) GDNF (mean change 3.021, adj *p* value < 0.001) and CCL4 (mean change 3.607, adi *p* value < 0.001; Figure 1A,B). According to the results of Silhouette method for number of clusters, hierarchical cluster analysis identified 3 clusters on day 0 [26]. Using one‐way ANOVA, the clusters differ in 46 proteins. The two non‐responders to dupilumab were grouped in Cluster 1, which was characterized by a low inflammatory signature. Only two patients were classified as non‐responders, and therefore these findings should be interpreted with caution. Cluster 3 was associated with the highest inflammatory signature. However, clusters did not differ significantly in SNOT‐22, NPS, SSIT‐12, or FeNO scores and there was no clear association with asthma, NERD, or allergy (Figure 1C). Based on previous studies, we classified patients into endotypes with predominant type 1 (IFN‐ γ), type 2 (IL5), and type 3 (IL‐17A) inflammation. Most patients (65.2%) exhibited a mixed inflammatory endotype (43.5% type 1/2/3, 17.4% type 2/3, 4.3% type 1/2). Patients with a pure type 3 (8.7%) and type 2 (13%) or an undefined endotypes (13%) were much less common (Figure 1D) [6, 23]. Overall, the type 2 endotype was most abundant (78.2%) followed by type 3 (69.6%) and type 1 (47.8%) (Figure 1D). Although the clusters did not show a relevant association with comorbidities like asthma or allergy, the percentage of allergic and asthmatic subjects in this cohort is very high and may affect the clustering.

### 3.2 Change of Nasal Secretion Inflammatory Markers Under Dupilumab Treatment

**FIGURE 2:**
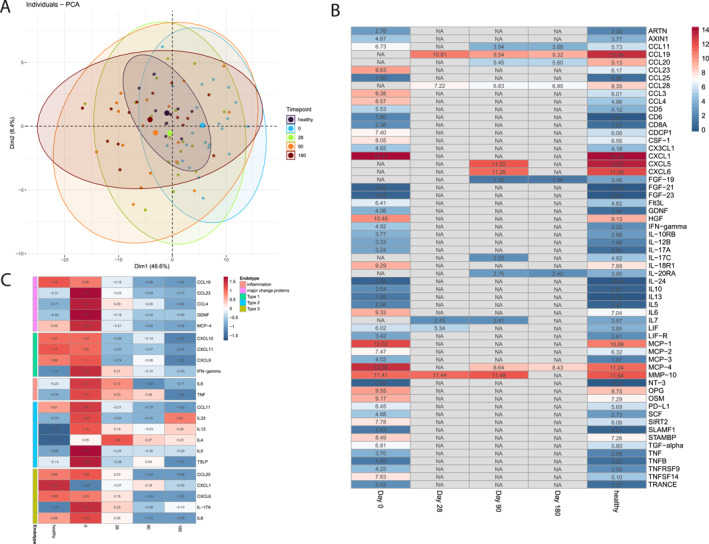
Nasal secretion cytokines under dupilumab therapy. (A) Principal component analysis (PCA) of all samples grouped according to timepoint (day 0, 28, 90, 180) and healthy controls. (B) Heatmap of means of only significant cytokines of nasal secretions samples on each timepoint compared to healthy controls. Only significant cytokines are displayed. NA means no significant change in the protein compared to healthy controls. (C) Heatmap of standardized means of all endotype specific cytokines at different timepoints. All cytokines are illustrated without significance testing.

The Principal Component Analysis (PCA) showed no clear clustering of the different time points under dupilumab treatment and healthy controls (Figure 2A). Compared to healthy individuals on day 28, after two injections with dupilumab, only five, after 90 days 12 and after 180 days seven proteins were significantly differentially expressed (Figure 2B). During therapy, changes in the hierarchical clusters were inconclusive. However, targeting IL‐4 and IL‐13 with dupilumab decreased the levels of not only type 2 cytokines, but also type 1 and 3 cytokines, as well as the proinflammatory cytokines IL‐6 and TNF, the CC chemokines, and the neurotrophic protein GDNF (Figure 2C). During therapy with dupilumab the proportion of patients with a mixed inflammatory endotype decreased to 21.5% in favor of patients with an undefined inflammatory endotype, which rose to 64.7% on day 90 after therapy start (Figure 1D). Both non‐responders initially had an undefined endotype, one showed no change and one changed to type 1/3 under therapy with dupilumab (Figure S1).

**FIGURE 3:**
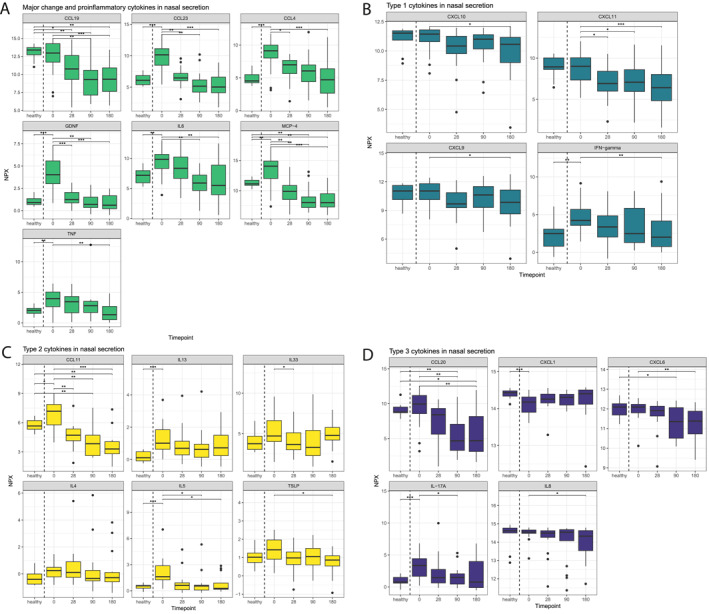
Cytokines concentrations in nasal secretion in CRSwNP. (A) Boxplots of the major change proteins and the proinflammatory cytokines IL‐6 and TNF. (B) Type 1 cytokines and chemokines, (C) Type 2 related cytokines and chemokines, (D) Type 3 cytokines and chemokines. Adjusted *p* values are illustrated with brackets. Only significant values are displayed. For comparison with healthy controls *t*‐test was used. For longitudinal analysis, paired *t*‐test was used. All statistical teste were corrected for multiple testing post‐hoc using Benjamini Hochberg method. *p* value * 0.05, ** 0.01, *** 0.001.

Individual cytokines of type 1, type 2, and type 3, as well as the proinflammatory cytokines IL‐6 and TNF‐α, the CC chemokines MCP4, CCL11, CCL23, CCL4 and the glial cell line–derived neurotrophic factor GDNF, all decreased significantly under therapy with dupilumab (Figure 3A–D). No significant changes in these cytokines were detected in serum samples except for the type 1 cytokines CXCL10 and CXCL11 as well as type 3 cytokine IL‐8 which increased significantly (Figure S2A–D).

### 3.3 Clinical Correlations

**FIGURE 4:**
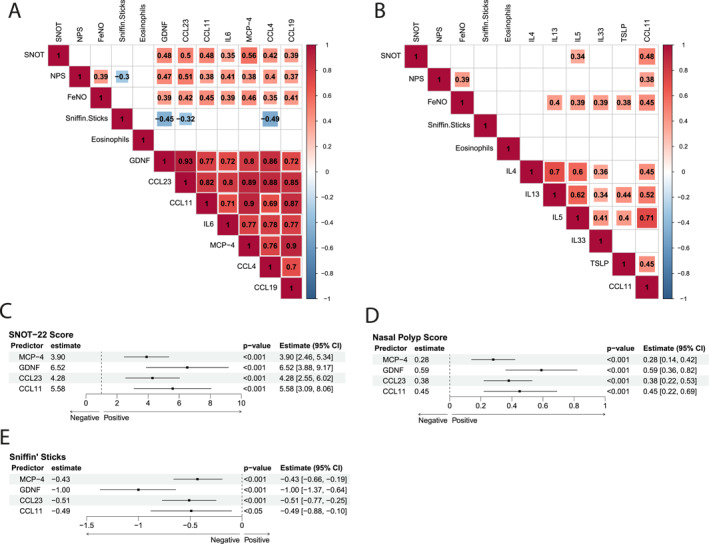
Correlation of clinical parameters and nasal secretion proteins. (A) Correlation plot of major change proteins under dupilumab treatment and clinical variables (SNOT‐22, NPS, FeNO, Sniffin‐Sticks, blood eosinophils). (B) Correlation plot of type 2 cytokines and chemokines. For correlation, Spearman's correlation was used and data from all timepoints. (C) Univariate linear regression of major change proteins (MCP‐4, GDNF, CCL23, CCL11) with SNOT‐22 score, (D) nasal polyp score, (E) 12‐item Sniffin’ stick score. Data of all timepoints under dupilumab treatment was used for analysis.

Spearman correlation analysis of clinical variables and proteins under dupilumab treatment across all follow‐ups showed a significant positive correlation between CCL23 and Nasal Polyp Score (Spearman's rho 0.513, FDR < 0.001), SNOT‐22 score (Spearman's rho 0.499, FDR < 0.001), SSIT‐12 (Spearman's rho −0.321, FDR 0.016) and exhaled NO fraction (Spearman's rho 0.42, FDR 0.003; Figure 4A). In addition, GDNF, and MCP‐4 correlated positively with SNOT‐22 score (GDNF: Spearman's rho 0.48, FDR 0.001), (MCP‐4: Spearman's rho 0.56, FDR < 0.001), NPS (GDNF: Spearman's rho 0.47, FDR 0.001), (MCP‐4: Spearman's rho 0.38, FDR 0.01) and FeNO (GDNF: Spearman's rho 0.39, FDR 0.008), (MCP‐4: Spearman's rho 0.46, FDR 0.001). Among the type 2 cytokines, only CCL11 correlated positively with SNOT‐22 scores (Spearman's rho 0.483, FDR < 0.001), NPS (Spearman's rho 0.377, FDR 0.003) and FeNO (Spearman's rho 0.454, FDR 0.001; Figure 4B). There were no protein expression levels correlating with blood eosinophila (Figure 4A,B). In a univariate linear regression model, MCP‐4, GDNF, CCL11, and CCL23 showed a significant influence on SNOT‐22, NPS and SSIT‐12 outcome (Figure 4C–E).

### 3.4 Potential Nasal Secretion Biomarkers for Disease Control and Therapy Response in Type 2 CRSwNP

**FIGURE 5:**
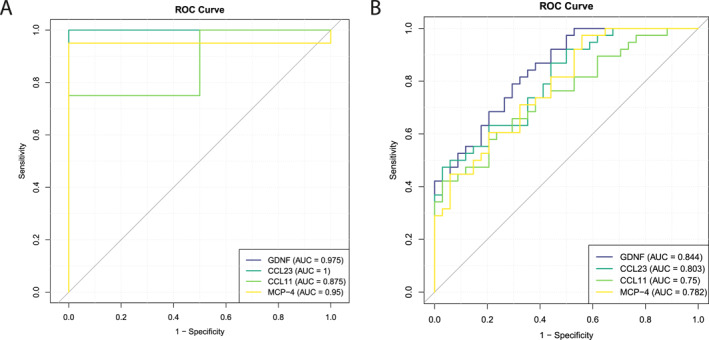
Receiver operating curve of major change proteins (GDNF, MCP‐4, CCL11, CCL23). (A) ROC curves of the major change proteins (GDNF, CCL23, CCL11, MCP‐4) predicting dupilumab therapy response after 3 or 6 months at baseline (day 0), with AUC values shown in the legend. Only baseline (day 0) values prior to dupilumab initiation were included in the analysis. (B) ROC curves of the major change proteins (GDNF, CCL23, CCL11, MCP‐4) predicting disease control, with AUC values shown in the legend. Since disease control represents a clinical status, data from all time points were included.

The most promising cytokines to reflect disease control of CRSwNP according to correlation analyses were GDNF, CCL23, CCL11, and MCP‐4. To assess the prediction of disease control in CRSwNP and response to dupilumab treatment, we conducted receiver‐operating curve (ROC) analyses with area under the curve (AUC). Disease control was defined based on EPOS20/EUFOREA expert opinion with a SNOT‐22 score below < 40 points and a total nasal polyp score of ≤ 2 points or hyposmia with a SSIT‐12 score of ≥ 7 points [24, 27]. Treatment response was defined as an improvement in the SNOT‐22 of ≥ 12 points according to the MCID and a decrease of the NPS of ≥ 1 point or increase of the SSIT‐12 of ≥ 2 points and above 7 points at 3 months or 6 months after treatment start. GDNF at baseline showed the best AUC with 0.975 for treatment response prediction at threshold of 1.61 (Sensitivity 95%, Specificity 100%) and 0.844 for disease control measured at each timepoint at a threshold of 1.84 NPX (Sensitivity 79%, Specificity 71%; Figure 5A,B).

## 4 Discussion

In this study, we investigated the inflammatory patterns of nasal secretions before and during therapy with dupilumab in patients with diffuse type 2 CRSwNP. Our findings indicate that most patients exhibit a mixed inflammatory pattern in nasal secretion and that therapeutic intervention targeting the type 2 pathway with dupilumab improved not only type 2, but also type 1 and type 3 inflammation. GDNF measured at each timepoint has emerged as a potential surrogate marker for disease control in patients with type 2 CRSwNP undergoing dupilumab therapy, and GDNF at baseline may also serve as a predictor of treatment response.

In line with previous studies, the nasal secretions of our cohort showed a very heterogeneous inflammatory pattern with the upregulation of 48 different proteins compared to healthy controls. As the healthy control group did not present with asthma or clinically relevant allergies, some of the observed differences in protein expression may also be influenced by comorbid asthma or allergy in the CRSwNP cohort, rather than CRSwNP alone. Different cluster analyses have been suggested with the aim of assigning protein patterns to a specific phenotype [6, 8, 12, 23]. In our cohort, a hierarchical unsupervised clustering of nasal secretion proteins revealed three clusters with varying degrees of inflammation. The non responders to dupilumab therapy were located in the same cluster with the lowest protein expression. However, a clear endotype‐phenotype classification was not possible. This was probably also due to the small number of patients for representative cluster analyses. Furthermore, 65% of the study population had comorbid allergies and 91% had asthma, reflecting the known association with type 2 CRSwNP. Although no significant association was observed between inflammatory clusters and asthma or allergy, the high prevalence of these comorbidities may still influence the observed inflammatory patterns. Therefore, the generalizability of our findings to populations with lower asthma prevalence may be limited, and studies in more heterogeneous cohorts are needed. The endotyping of nasal secretions by signature cytokines (IFN‐γ, IL‐5, IL‐17A) for the type 1, type 2 and type 3 inflammatory endotype revealed a mixed inflammatory pattern in most of the patients. Most probably, the protein pattern of nasal secretion is influenced by cofactors such as bacteria, viruses, fungi and allergies. An increased susceptibility for infections and allergic sensitization might be induced by the disrupted epithelial barrier in local type 2 inflammation [28, 29, 30]. In addition, the increased IL‐4 in the context of type 2 inflammation leads to a decrease of neutrophil NET formation and neutrophil migration leading to impaired phagocytic capacity which may also increase the risk of infection [31, 32, 33, 34]. IL‐4 can also promote the differentiation of M2 macrophages, followed by tissue remodeling and the secretion of anti‐inflammatory cytokines, such as TGF‐β and IL‐10 [35, 36, 37, 38]. M2 macrophages can also prolong survival of bacteria and viruses, promoting type 1 and type 3 inflammation in the nasal secretion [39]. These findings indicate that type 2 CRSwNP cannot be clearly diagnosed using nasal secretion endotyping.

In our study, treatment with the anti‐IL‐4Rα‐antibody dupilumab was associated not only with a decrease of type 2 but also with a decrease of type 1, and type 3 cytokines. The group with a mixed inflammatory endotype changed in favor of the group with an undefined type of inflammation. Treatment with dupilumab resulted in a significant reduction in type 2, with type 3 being the most prevalent secretory endotype after 180 days of treatment (42.8%). There was a shift in the inflammation pattern toward levels comparable to those observed in the healthy control group. These results are in line with the findings in atopic dermatitis, where a reduction of type 2 cytokines under dupilumab was observed in lesional and non‐lesional skin [21]. Even in alopecia areata, reduced Th2‐related chemokines (MCP‐4, eotaxin‐2) and a decrease in Th1‐related markers (CXCL10/CXCL11) were observed during dupilumab treatment [40]. As recently shown, dupilumab treatment in CRSwNP had an influence on nasal mucosa microbiota with an increase in protective commensal genera and with restoration of the epithelial barrier [17, 18]. These findings indicate that blocking IL‐4 and IL‐13 in CRSwNP not only reduces the local type 2 inflammation markers but also improves restoration of the nasal mucosal epithelial barrier, increases the protective microbiota and reduces the risk of superinfections that may be associated with type 1 and type 3 inflammation. Therefore, the determination of the inflammatory endotype of nasal secretions in type 2 CRSwNP seems to be influenced by multiple co‐factors and the underlying type 2 inflammation often is not adequately represented in the nasal secretion. Accordingly, type 1, 2, and 3 inflammation‐specific cytokines appear to be too unreliable to diagnose and monitor type 2 CRSwNP.

GDNF and CCL23, the cytokines with the highest expression in type 2 CRSwNP as well as the chemokines MCP‐4 and CCL11 with the highest mean change on day 28 under therapy with dupilumab revealed as the best correlation markers with clinical SNOT‐22‐, Nasal Polyp‐ and Sniffin’Stick score. CCL11/Eotaxin‐1 is a chemoattractant for eosinophils and elevated in eosinophilic CRSwNP and in nasal polyp tissue [41, 42]. Its expression by epithelial cells and fibroblasts can be promoted by the alarmin TSLP as well as by IL‐4 and IL‐13 via the STAT6 pathway [43, 44, 45, 46, 47, 48, 49]. MCP‐4 (CCL13) attracts monocytes, Th2 cells, eosinophilic and basophil granulocytes. It can be highly expressed by M2‐macrophages in nasal polyps [35, 36, 38, 42, 50]. Elevated levels of CCL23 in nasal polyps of CRSwNP patients were described in previous studies [51, 52]. Induced by the Th2 cytokines IL‐4 and IL‐13 and produced by eosinophils, CCL23 is a chemoattractant for monocytes, dendritic cells and lymphocytes [52, 53, 54]. GDNF (glial‐derived neurotrophic factor) is a neurotrophin and secreted mostly by microglia in the central nervous system but also in the lungs [55]. GDNF is a member of TGF‐β superfamily and affects airway remodeling and can have a procontractile effect on airway smooth muscle cells [56, 57]. GDNF binds to the GFRα1/RET receptor and drives MAPK/ERK1/2 and PI3K/AKT signaling pathways in primary human nasal epithelial cells (HNECs) promoting cell proliferation as recently shown [58]. MAPK and PI3K/Akt were also significantly enriched in transcriptomic analysis following GDNF stimulation in HNECs. Nasal tissue with high levels of GDNF showed epithelial thickening and basal cell hyperplasia [58, 59]. We hypothesize that IL‐4 directly throuth STAT6 pathway or via polarization of M2 macrophages and the subsequent TGF‐beta secretion induce GDNF production. Dupilumab may therefore inhibit the function of IL‐4 and decrease M2 polarization and subsequently TGF‐beta secretion. In a model for diagnosing type 2 CRSwNP, Hou et al. and Morgenstern et al. identified GDNF in nasal secretion as a promising biomarker for type 2 CRSwNP [8, 60]. All four markers showed significant correlation with clinical variables and a good AUC in assessing disease control in type 2 CRSwNP under dupilumab. GDNF demonstrated to be the most effective marker for predicting the response to dupilumab therapy before treatment starts, as well as for monitoring disease control in type 2 CRSwNP. Therefore, according to our results and the recent literature, the neurotrophic protein GDNF rather than a classical Th2 mediator or a distinct endotype defined by cluster analysis, revealed as the best marker for evaluating therapeutic response and disease control in type 2 CRSwNP.

Interestingly, we observed a significant increase in type 1 (CXCL10, CXCL11) and type 3 (IL‐8) cytokines in the peripheral blood. This is in line with previous results in bullous pemphigoid treated with dupilumab [61]. Th1/Th17‐mediated skin and musculoskeletal disorders as adverse events of dupilumab in CRSwNP like psoriasis or seronegative arthritis have been previously reported [62, 63, 64]. In our cohort, the increase in type 1 and type 3 cytokines observed in the blood was not associated with clinical symptoms. Although its clinical relevance has yet to be proven, systemic dupilumab treatment seems to have an immunomodulatory effect on peripheral blood. Therefore, the dosage should be as high as necessary and as low as possible. This finding also confirms the importance of researching biomarkers that indicate response to treatment and disease control under systemic biological therapies. Once steady state and homeostasis have been achieved, tapering dupilumab therapy by extending treatment intervals could be considered [65].

This study has several limitations. The collection of nasal secretion is associated with many confounders such as placement of the surgical patty and dilution, which may reduce reproducibility [66]. The cohort size was relatively small and underpowered for cluster analysis, and included only two non‐responders, thereby limiting conclusions regarding predictors of dupilumab treatment response and underlying inflammatory endotypes. Furthermore, the study population had a high percentage of comorbid asthma or allergy patients, limiting the clustering solely due to CRSwNP. The endotyping approach was defined based on the healthy control samples collected in this study. However, the limited number of healthy controls (*n* = 10), compared with previous studies, may affect the robustness of the 95th percentile cut‐off. In addition, adherence issues led to missing clinical data.

## 5 Conclusion

The nasal secretion of patients with type 2 CRSwNP is associated with a mixed inflammatory signature. Accordingly, the reliability of endotyping CRSwNP using nasal secretion seems to be limited. Dupilumab improves all inflammatory endotypes in the nasal mucosal inflammation, helping to restore a healthy steady state. We identified GDNF as potential marker to predict therapeutic response and to monitor disease control in type 2 CRSwNP during dupilumab therapy.

## Author Contributions

**Fabio S. Ryser:** writing – original draft, methodology, project administration, formal analysis, conceptualization, investigation, visualization, data curation, writing – review and editing. **Tina Mauthe:** investigation, writing – review and editing. **Catrin Bruhlmann:** writing – review and editing, investigation. **Michael B. Soyka:** conceptualization, investigation, supervision, writing – review and editing, methodology. **Urs C. Steiner:** conceptualization, investigation, funding acquisition, writing – original draft, supervision, methodology.

## Funding

This work was supported by unrestricted institutional grant of UCS.

## Conflicts of Interest

M. B. S. is a consultant for Sanofi, GSK, Novartis, Astra Zeneca, and M. S. D. unrelated to this study. U. C. S. is a consultant for Sanofi, GSK, TAKEDA, Otsuka and Astra Zeneca unrelated to this study. The other authors reported no conflicts of interest.

## Supporting information


**Figure S1:** Inflammatory endotypes in the nasal secretion under dupilumab therapy. (A) Endotypes of individual patients at each time point during dupilumab treatment, shown as pie charts. Type 1/2/3 endotypes were defined based on increased levels of IFN‐γ, IL‐5, and IL‐17A (above the 95th percentile compared to controls).

**Figure S2:** Cytokines concentrations in blood serum samples in CRSwNP. (A) Boxplots of proteins in the serum with the highest mean change from day 0,28, and 90 under dupilumab therapy in nasal secretions. (B) Type 1 and proinflammatory (IL‐6, TNF) cytokines and chemokines, (C) Type 2 cytokines and chemokines, (D) Type 3 cytokines and chemokines. *p* values are illustrated with brackets. Only significant values are displayed. For comparison with healthy controls *t*‐test was used. For longitudinal analysis, paired *t*‐test was used. All statistical teste were corrected for multiple testing post‐hoc using Benjamini Hochberg method. *p* value *0.05, **0.01, ***0.001.

**Table S1:** Proteins included in the Olink Inflammation Target 96 panel. Protein targets were measured using Olink Proximity Extension Assay (PEA) technology. All values are reported in Normalized Protein Expression (NPX) units. LLOQ: lower limit of quantification; LOD: limit of detection; ULOQ: upper limit of quantification.

## Data Availability

The data that support the findings of this study are available from the corresponding author upon reasonable request.
