# Supplementary material for: Improvement of Mixed Inflammatory Environment in Nasal Secretions of Diffuse Type 2 Chronic Rhinosinusitis With Nasal Polyps Under Dupilumab
Source: Clin Transl Allergy. 2026 Jun 3;16(6):e70180. doi: 10.1002/clt2.70180 (PMC13239652; doi:10.1002/clt2.70180)

Supp. Figure 1

A

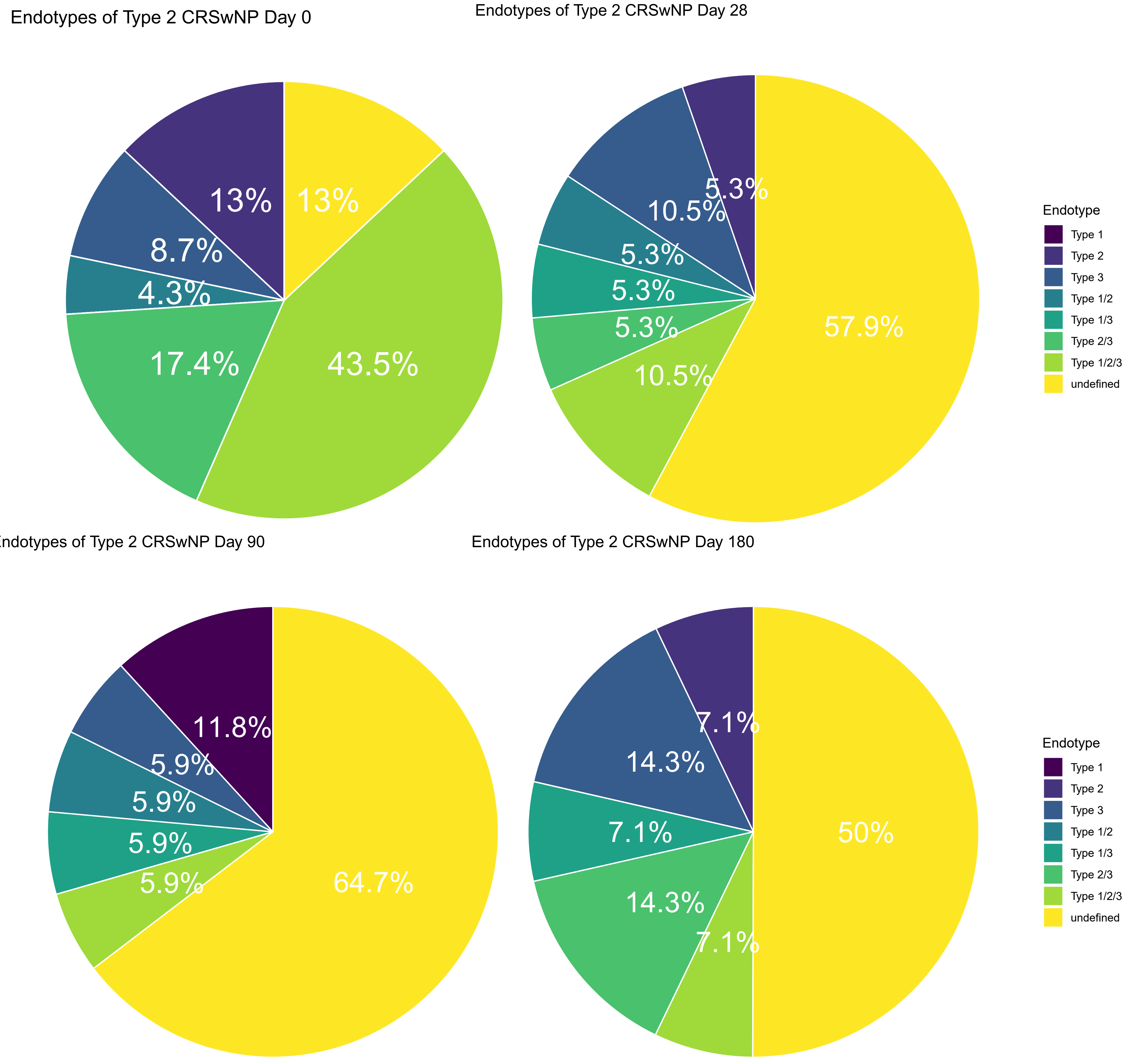

Supplement: Supplementary file 1 — Figure S1: Inflammatory endotypes in the nasal secretion under dupilumab therapy. (A) Endotypes of individual patients at each time point during dupilumab treatment, shown as pie charts. Type 1/2/3 endotypes were defined based on increased levels of IFN‐γ, IL‐5, and IL‐17A (above the 95th percentile compared to controls). [file CLT2-16-e70180-s001.pdf]
