# Supplementary material for: Improvement of Mixed Inflammatory Environment in Nasal Secretions of Diffuse Type 2 Chronic Rhinosinusitis With Nasal Polyps Under Dupilumab
Source: Clin Transl Allergy. 2026 Jun 3;16(6):e70180. doi: 10.1002/clt2.70180 (PMC13239652; doi:10.1002/clt2.70180)

Supp. Figure 2

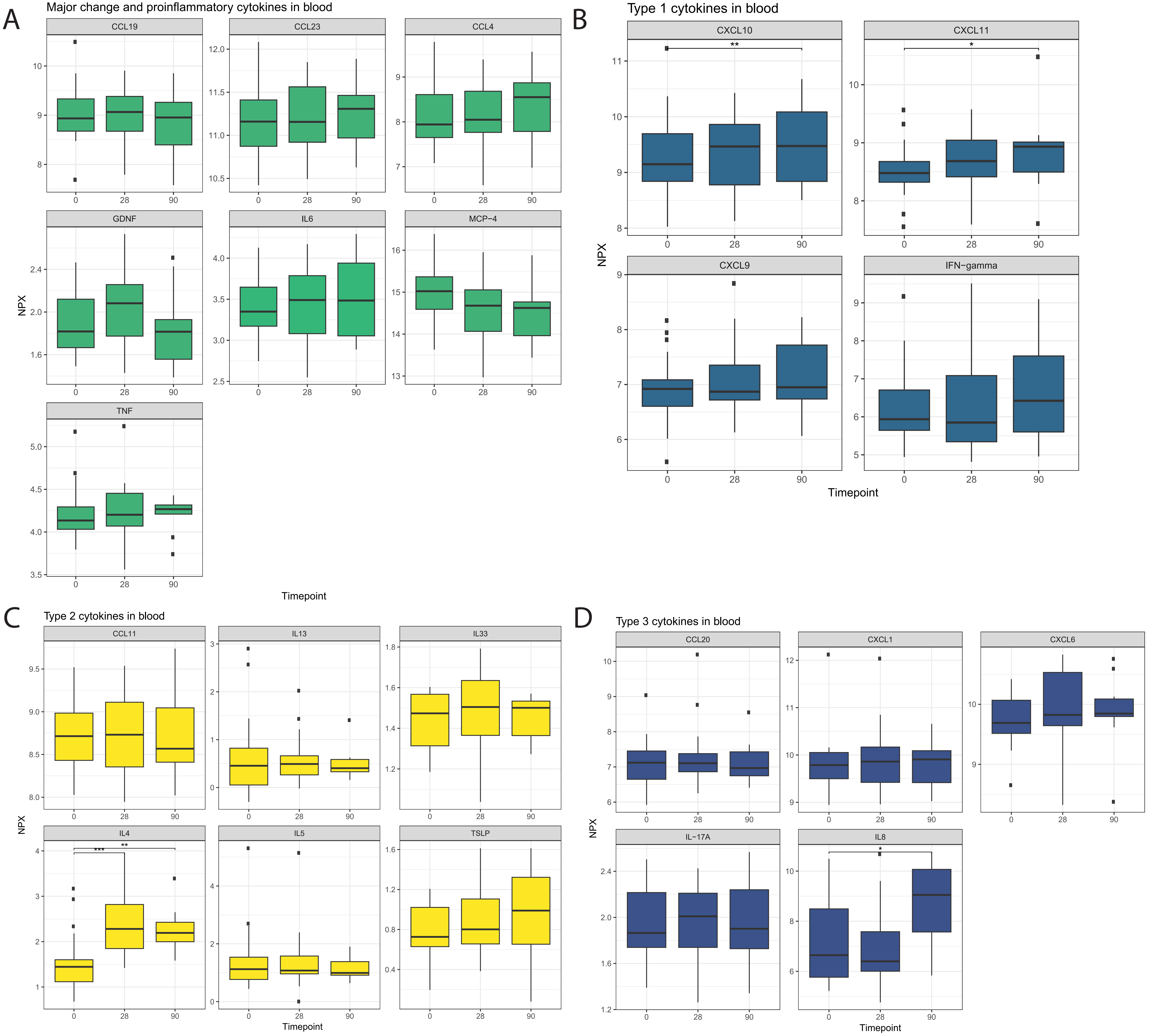

Supplement: Supplementary file 2 — Figure S2: Cytokines concentrations in blood serum samples in CRSwNP. (A) Boxplots of proteins in the serum with the highest mean change from day 0,28, and 90 under dupilumab therapy in nasal secretions. (B) Type 1 and proinflammatory (IL‐6, TNF) cytokines and chemokines, (C) Type 2 cytokines and chemokines, (D) Type 3 cytokines and chemokines. p values are illustrated with brackets. Only significant values are displayed. For comparison with healthy controls t‐test was used. For longitudinal analysis, paired t‐test was used. All statistical teste were corrected for multiple testing post‐hoc using Benjamini Hochberg method. p value *0.05, **0.01, ***0.001. [file CLT2-16-e70180-s003.pdf]
