# Supplementary material for: Improvement of Mixed Inflammatory Environment in Nasal Secretions of Diffuse Type 2 Chronic Rhinosinusitis With Nasal Polyps Under Dupilumab
Source: Clin Transl Allergy. 2026 Jun 3;16(6):e70180. doi: 10.1002/clt2.70180 (PMC13239652; doi:10.1002/clt2.70180)
Supplement: Supplementary file 3 — Table S1: Proteins included in the Olink Inflammation Target 96 panel. Protein targets were measured using Olink Proximity Extension Assay (PEA) technology. All values are reported in Normalized Protein Expression (NPX) units. LLOQ: lower limit of quantification; LOD: limit of detection; ULOQ: upper limit of quantification. [file CLT2-16-e70180-s002.docx]

Supplementary Table 1:

| **UniProt ID** | **Gene** | **Protein name** | **LOD** | **LLOQ** | **ULOQ** | **Hook** | **log10 Range** |
| --- | --- | --- | --- | --- | --- | --- | --- |
| P00813 | ADA | Adenosine deaminase | 0.48 | 0.48 | 31250 | 12500 | 4.8 |
| Q5T4W7 | ARTN | Artemin | 0.24 | 0.48 | 31250 | 62500 | 4.8 |
| O15169 | AXIN1 | Axin-1 | 61 | 61 | 62500 | 250000 | 3 |
| P01138 | NGF | Beta-nerve growth factor | 0.48 | 0.48 | 15625 | 31250 | 4.5 |
| Q14790 | CASP8 | Caspase-8 | 0.48 | 0.48 | 31250 | 62500 | 4.8 |
| Q99616 | CCL13 | C-C motif chemokine 13 | 0.24 | 0.24 | 1953 | 7812 | 3.9 |
| Q99731 | CCL19 | C-C motif chemokine 19 | 15 | 15 | 31250 | 62500 | 3.3 |
| P13500 | CCL2 | C-C motif chemokine 2 | 0.03 | 0.03 | 1953 | 3906 | 4.8 |
| P78556 | CCL20 | C-C motif chemokine 20 | 7.6 | 7.6 | 15625 | 15625 | 3.3 |
| P55773 | CCL23 | C-C motif chemokine 23 | 31 | 31 | 31250 | 62500 | 3 |
| O15444 | CCL25 | C-C motif chemokine 25 | 3.8 | 3.8 | 62500 | 125000 | 4.2 |
| Q9NRJ3 | CCL28 | C-C motif chemokine 28 | 61 | 122 | 1000000 | 1000000 | 3.9 |
| P10147 | CCL3 | C-C motif chemokine 3 | 0.06 | 0.06 | 488 | 977 | 3.9 |
| P13236 | CCL4 | C-C motif chemokine 4 | 1.9 | 1.9 | 31250 | 62500 | 4.2 |
| P80098 | CCL7 | C-C motif chemokine 7 | 0.48 | 0.48 | 1953 | 3906 | 3.6 |
| P80075 | CCL8 | C-C motif chemokine 8 | 0.06 | 0.06 | 3906 | 7812 | 4.8 |
| Q9H5V8 | CDCP1 | CUB domain-containing protein 1 | 0.12 | 0.12 | 7812 | 31250 | 4.8 |
| P02778 | CXCL10 | C-X-C motif chemokine 10 | 7.6 | 7.6 | 15625 | 31250 | 3.3 |
| O14625 | CXCL11 | C-X-C motif chemokine 11 | 7.6 | 31 | 15625 | 15625 | 2.7 |
| P42830 | CXCL5 | C-X-C motif chemokine 5 | 0.95 | 0.95 | 7812 | 15625 | 3.9 |
| P80162 | CXCL6 | C-X-C motif chemokine 6 | 7.6 | 31 | 15625 | 31250 | 2.7 |
| Q07325 | CXCL9 | C-X-C motif chemokine 9 | 0.95 | 0.95 | 3906 | 7812 | 3.6 |
| P28325 | CST5 | Cystatin-D | 1.9 | 1.9 | 15625 | 31250 | 3.9 |
| Q8NFT8 | DNER | Delta and Notch-like epidermal growth factor-related receptor | 0.95 | 1.9 | 31250 | 62500 | 4.2 |
| P51671 | CCL11 | Eotaxin | 3.8 | 3.8 | 31250 | 62500 | 3.9 |
| Q13541 | EIF4EBP1 | Eukaryotic translation initiation factor 4E-binding protein 1 | NA | NA | NA | NA | NA |
| O95750 | FGF19 | Fibroblast growth factor 19 | 7.6 | 7.6 | 15625 | 31250 | 3.3 |
| Q9NSA1 | FGF21 | Fibroblast growth factor 21 | 31 | 31 | 62500 | 500000 | 3.3 |
| Q9GZV9 | FGF23 | Fibroblast growth factor 23 | 122 | 122 | 62500 | 62500 | 2.7 |
| P12034 | FGF5 | Fibroblast growth factor 5 | 1.9 | 1.9 | 31250 | 125000 | 4.2 |
| P49771 | FLT3LG | Fms-related tyrosine kinase 3 ligand | 0.01 | 0.01 | 977 | 3906 | 4.8 |
| P78423 | CX3CL1 | Fractalkine | 15.3 | 15.3 | 15625 | 31250 | 3 |
| P39905 | GDNF | Glial cell line-derived neurotrophic factor | 0.01 | 0.01 | 1953 | 3906 | 5.1 |
| P09341 | CXCL1 | Growth-regulated alpha protein | 3.8 | 7.6 | 15625 | 15625 | 3.3 |
| P14210 | HGF | Hepatocyte growth factor | 7.6 | 7.6 | 125000 | 125000 | 3.9 |
| P01579 | IFNG | Interferon gamma | 0.24 | 0.24 | 15625 | 31250 | 4.8 |
| P01583 | IL1A | Interleukin-1 alpha | 0.48 | 0.95 | 31250 | 125000 | 4.5 |
| P22301 | IL10 | Interleukin-10 | 0.48 | 0.48 | 62500 | 125000 | 5.1 |
| Q13651 | IL10RA | Interleukin-10 receptor subunit alpha | 3.8 | 7.6 | 250000 | 500000 | 4.5 |
| Q08334 | IL10RB | Interleukin-10 receptor subunit beta | 0.12 | 0.12 | 1953 | 3906 | 4.2 |
| P29460 | IL12B | Interleukin-12 subunit beta | 0.12 | 0.12 | 3906 | 3906 | 4.5 |
| P35225 | IL13 | Interleukin-13 | 7.6 | 7.6 | 62500 | 500000 | 3.9 |
| Q13261 | IL15RA | Interleukin-15 receptor subunit alpha | 0.95 | 0.95 | 7812 | 15625 | 3.9 |
| Q16552 | IL17A | Interleukin-17A | 3.8 | 7.6 | 62500 | 62500 | 3.9 |
| Q9P0M4 | IL17C | Interleukin-17C | 31 | 31 | 125000 | 500000 | 3.3 |
| Q14116 | IL18 | Interleukin-18 | 0.06 | 0.06 | 15625 | 15625 | 5.4 |
| Q13478 | IL18R1 | Interleukin-18 receptor 1 | 0.06 | 0.06 | 7812 | 15625 | 5.1 |
| P60568 | IL2 | Interleukin-2 | 30.5 | 30.5 | 1000000 | 1000000 | 4.5 |
| P14784 | IL2RB | Interleukin-2 receptor subunit beta | 15 | 31 | 150000 | 1000000 | 3.9 |
| Q9NYY1 | IL20 | Interleukin-20 | 7.6 | 15 | 62500 | 125000 | 3.6 |
| Q9UHF4 | IL20RA | Interleukin-20 receptor subunit alpha | 1.9 | 1.9 | 125000 | 125000 | 4.8 |
| Q8N6P7 | IL22RA1 | Interleukin-22 receptor subunit alpha-1 | 0.24 | 0.24 | 3906 | 15625 | 4.2 |
| Q13007 | IL24 | Interleukin-24 | 1.9 | 3.8 | 31250 | 125000 | 3.9 |
| O95760 | IL33 | Interleukin-33 | 3.8 | 3.8 | 31250 | 125000 | 3.9 |
| P05112 | IL4 | Interleukin-4 | 0.24 | 0.24 | 7812 | 15625 | 4.5 |
| P05113 | IL5 | Interleukin-5 | 3.8 | 3.8 | 15625 | 62500 | 3.6 |
| P05231 | IL6 | Interleukin-6 | 0.12 | 0.12 | 3906 | 15625 | 4.5 |
| P13232 | IL7 | Interleukin-7 | 0.24 | 0.24 | 7812 | 15625 | 4.5 |
| P10145 | CXCL8 | Interleukin-8 | 0.03 | 0.03 | 3906 | 7812 | 5.1 |
| P03956 | MMP1 | Interstitial collagenase | 1.9 | 3.8 | 15625 | 31250 | 3.6 |
| P21583 | KITLG | Kit ligand | 1.9 | 3.8 | 15625 | 31250 | 4 |
| P15018 | LIF | Leukemia inhibitory factor | 3.8 | 7.6 | 15625 | 31250 | 3.3 |
| P42702 | LIFR | Leukemia inhibitory factor receptor | 30.5 | 15.3 | 62500 | 250000 | 3.3 |
| P01374 | LTA | Lymphotoxin-alpha | 0.24 | 0.48 | 15625 | 15625 | 4.5 |
| P09603 | CSF1 | Macrophage colony-stimulating factor 1 | 0.004 | 0.01 | 1953 | 3906 | 5.4 |
| Q8IXJ6 | SIRT2 | NAD-dependent protein deacetylase sirtuin-2 | 7.6 | 15.3 | 62500 | 250000 | 3.6 |
| Q9BZW8 | CD244 | Natural killer cell receptor 2B4 | 0.06 | 0.06 | 7812 | 15625 | 5.1 |
| P20783 | NTF3 | Neurotrophin-3 | 0.12 | 0.12 | 3906 | 7812 | 4.5 |
| Q99748 | NRTN | Neurturin | 3.9 | 7.6 | 15625 | 62500 | 3.3 |
| P13725 | OSM | Oncostatin-M | 0.03 | 0.03 | 977 | 3906 | 4.5 |
| Q9NZQ7 | CD274 | Programmed cell death 1 ligand 1 | 3.8 | 3.8 | 500000 | 1000000 | 5.1 |
| P80511 | S100A12 | Protein S100-A12 | 122 | 122 | 500000 | 1000000 | 3.6 |
| P01135 | TGFA | Protransforming growth factor alpha | 0.48 | 0.48 | 3906 | 31250 | 3.9 |
| Q13291 | SLAMF1 | Signaling lymphocytic activation molecule | 31 | 31 | 1000000 | 1000000 | 4.5 |
| O95630 | STAMBP | STAM-binding protein | 7.6 | 7.6 | 31250 | 62500 | 3.6 |
| P09238 | MMP10 | Stromelysin-2 | 0.95 | 0.95 | 15625 | 62500 | 4.2 |
| P50225 | SULT1A1 | Sulfotransferase 1A1 | 244 | 244 | 125000 | 500000 | 2.7 |
| P30203 | CD6 | T-cell differentiation antigen CD6 | 0.24 | 0.24 | 7812 | 31250 | 4.2 |
| P06127 | CD5 | T-cell surface glycoprotein CD5 | 0.06 | 0.12 | 3906 | 15625 | 4.5 |
| P01732 | CD8A | T-cell surface glycoprotein CD8 alpha chain | NA | NA | NA | NA | NA |
| Q969D9 | TSLP | Thymic stromal lymphopoietin | 3.8 | 3.8 | 15625 | 62500 | 3.6 |
| P01137 | TGFB1 | Transforming growth factor beta-1 proprotein | 0.24 | 0.48 | 15625 | 15625 | 4.5 |
| P01375 | TNF | Tumor necrosis factor | 0.95 | 0.95 | 31250 | 500000 | 4.5 |
| P50591 | TNFSF10 | Tumor necrosis factor ligand superfamily member 10 | 0.95 | 0.95 | 31250 | 31250 | 4.5 |
| O14788 | TNFSF11 | Tumor necrosis factor ligand superfamily member 11 | 3.8 | 3.8 | 31250 | 125000 | 3.9 |
| O43508 | TNFSF12 | Tumor necrosis factor ligand superfamily member 12 | 1.9 | 1.9 | 125000 | 500000 | 4.8 |
| O43557 | TNFSF14 | Tumor necrosis factor ligand superfamily member 14 | 0.95 | 1.9 | 15625 | 31250 | 3.9 |
| O00300 | TNFRSF11B | Tumor necrosis factor receptor superfamily member 11B | 0.24 | 0.48 | 31250 | 62500 | 4.8 |
| P25942 | CD40 | Tumor necrosis factor receptor superfamily member 5 | 0.01 | 0.01 | 3906 | 15625 | 5.4 |
| Q07011 | TNFRSF9 | Tumor necrosis factor receptor superfamily member 9 | 0.03 | 0.03 | 3906 | 3906 | 5.1 |
| P00749 | PLAU | Urokinase-type plasminogen activator | 0.12 | 0.12 | 7812 | 15625 | 4.8 |
| P15692 | VEGFA | Vascular endothelial growth factor A | 0.06 | 0.06 | 7812 | 15625 | 5.1 |

LOD: limits of detection, LLOQ: lower limits of quantification, ULOQ: upper limits of quantification
